# COVID-19 Pandemic Experiences and Hazardous Alcohol Use: Findings of Higher and Lower Risk in a Heavy-Drinking Midwestern State

**DOI:** 10.3390/ijerph22081230

**Published:** 2025-08-07

**Authors:** Justinian Wurtzel, Paul A. Gilbert, Loulwa Soweid, Gaurab Maharjan

**Affiliations:** 1 Bureau of HIV, STI, and Hepatitis, Division of Public Health, Iowa Department of Health and Human Services, Des Moines, IA 50319, USA; justinian.wurtzel@hhs.iowa.gov; 2 Department of Community and Behavioral Health, College of Public Health, University of Iowa, Iowa City, IA 52246, USA; loulwa-soweid@uiowa.edu (L.S.); gaurab-maharjan@uiowa.edu (G.M.)

**Keywords:** COVID-19, stress, alcohol drinking, binge drinking, social support

## Abstract

This study assessed whether COVID-19 pandemic experiences were associated with excessive alcohol use during the first year of the pandemic in Iowa, a heavy-drinking midwestern US state. We analyzed survey data from 4047 adult residents of Iowa collected in August 2020, focusing on three pandemic-related stressors (e.g., emotional reactions to the pandemic; disruption of daily activities; and financial hardship) and salient social support. Using multiple logistic regression, we tested correlates of increased drinking, heavy drinking, and binge drinking, controlling for demographic characteristics and health status. We found that nearly half (47.6%) of respondents did not change their drinking compared to before the pandemic; however, 12.4% of respondents reported increasing their drinking and 5.3% reported decreasing their drinking. Emotional reactions to the pandemic and disruption of daily activities were associated with higher odds of increased drinking, and rurality was associated with lower odds of increased drinking. No pandemic-related stressor was associated with heavy or binge drinking, but social support was associated with lower odds of binge drinking. Thus, we concluded that some pandemic-related stressors may explain increased drinking but not heavy or binge drinking. Understanding the nuances of alcohol use can inform preventive interventions, policy decisions, and preparations for future catastrophic events.

## 1. Introduction

The COVID-19 pandemic introduced a range of novel stressors, particularly due to changes in physical and social environments, that could have undermined people’s well-being. For example, protective measures such as stay-at-home orders and social distancing requirements may have had direct negative impacts through loss of income or social isolation as well as indirect negative impacts by triggering maladaptive coping behaviors, including substance use. In particular, alcohol use is both a theoretically predicted and an empirically documented coping response to many stressors [1,2,3,4,5,6]. Coping is one of four widely recognized motivations of drinking [7], and recent research with college students in the United States (US) found that drinking to cope partially explained the association between COVID-19 anxiety and alcohol-related problems [8]. In addition, social isolation and loneliness have been long regarded as risk-factors for alcohol use disorder [9,10,11].

Although some early reports showed increased drinking internationally and in the US during the first year of the COVID-19 pandemic [12,13,14], it appears that changes in alcohol consumption were not uniform. Some studies found evidence of both increasing and decreasing alcohol consumption, while others found significant decreases in alcohol use during the pandemic compared to prior [15]. Notably, a Canadian study identified COVID-19 stressors related to alcohol outcomes [16]. Depression, social disconnection, and parenting stress (e.g., having children at home) were associated with increased alcohol consumption, and living alone was associated with greater solitary drinking. These factors were further associated with increased drinking as a coping motive, which in turn was associated with increased alcohol problems. In addition, changes in drinking behaviors often varied by population sub-groups, such that some groups were more likely to increase drinking than others. Those differential responses were most often seen by age, gender, educational attainment, and home context, such as minor children in a household or young adults living with family members [13,17,18,19,20,21]. The variability in findings may be due to differences in drinking motives, which reflect distinct psychological needs that drive alcohol consumption [7]. Some individuals may rely on alcohol to cope with negative emotions and hence could have increased drinking during the pandemic, while those who drink as a custom or as a normative social behavior may have decreased drinking.

Further analyses have extended the above-mentioned descriptive literature. Notably, research has identified psychosocial correlates of changes in drinking behavior during the pandemic. Foremost—and as expected—there have been reports that pandemic-related stress was associated with alcohol use [17,22]. Studies that assessed drinking motives found that coping (e.g., tension reduction/relaxation) was associated with drinking [16,23,24]. In addition, some studies have found moderation by gender, such that COVID-19-related stress was more strongly associated with drinking among women than men [25,26]. Finally, some pandemic restrictions, such as lockdown or stay-at-home orders, may be associated with a greater likelihood of binge drinking during the pandemic [27]. While reducing COVID-19 risk exposures, these measures could have had the unintended consequence of exacerbating social or perceived isolation (i.e., loneliness) and psychological distress, which in turn led to more alcohol use as a coping response [28].

An additional factor that should be considered is geographical context. Epidemiological data have shown that drinking patterns vary across the US, and the Midwest is a particularly heavy-drinking region [29]. During the first year of the pandemic, Iowa ranked second highest in the nation for binge drinking prevalence [30]. Compounding concerns about excessive drinking, several state policy changes expanded access to alcohol during the pandemic. Framed as strategies to support the hospitality and food service sectors, a governor’s order initially allowed carry-out of alcoholic beverages from restaurants and bars, curbside pick-up of alcohol orders from retailers, and home delivery of alcohol through third-party services [31]. Subsequently, the Iowa legislature made these changes permanent [32,33]. Alcohol prevention specialists raised concerns, as restricting availability is a consistent policy recommendation to reduce excessive drinking and related problems [34,35,36]. Not surprisingly, Iowa recorded record-breaking liquor sales during the pandemic [34,35]. Contemporaneously, there was increasing attention to alcohol-related deaths and recent evidence shows that alcohol-related mortality increased by 58% from 2018 to 2022 [34,35,36,37]. In contrast, many countries in Europe saw closures of liquor stores, bars and pubs, and other drinking environments (e.g., hotels), with overall declines in average alcohol consumption, barring unique exceptions [38,39].

Given the high level of alcohol consumption in Iowa prior to the pandemic as well as contemporaneous indications of concern, such as sharp increases in liquor sales during the first year of the pandemic, we investigated the associations of a set of pandemic experiences—three types of stressors and pandemic-related social support—on drinking among adults. We hypothesized that there would be a positive association between the stressors and alcohol use and a negative association between social support and alcohol use.

## 2. Methods

Details of the parent study have been previously reported [40]. Briefly, a cross-sectional survey was fielded in August 2020 to assess behavioral changes during the COVID-19 pandemic in Iowa. Respondents were selected from 2018 county voter registration lists via stratified random sampling, with oversampling of residents from six rural counties. Inclusion criteria required respondents to be adults (age 18–100 years) who were able to communicate in English or Spanish. In total, 10,009 individuals were selected from the voter registration lists and received a pre-notification postcard about the study. One week later, a packet was mailed to the sampled individuals, which included English and Spanish versions of an informed consent letter, English and Spanish versions of the survey, a $5 cash incentive, and information about an option to complete the questionnaire online. A waiver of documentation of consent was granted by the University of Iowa IRB due to the survey modality.

Of the sampled individuals, 4048 (40.4%) completed the questionnaire, of which 597 (14.7%) were completed online. No surveys were completed in Spanish. A detailed flowchart showing the parent study’s sample derivation has been previously published [40]. In preparing the dataset for this analysis, we discovered that one participant had insufficient data to count as a completed survey; thus, the final analytic sample consisted of 4047 individuals.

Alcohol outcomes were measured by four items drawn from a collaborative survey about COVID-19 pandemic experiences [41]. Respondents answered a categorical question about changes in their alcohol use since the onset of the pandemic, with response options of drinking more, drinking less, or drinking the same as before the pandemic, as well as neither drinking before nor during the pandemic. From those responses we created a binary indicator of increased alcohol use (any vs. none) for analysis. The questionnaire also included items about the frequency of past 30-day drinking (i.e., count of days), the quantity consumed on a typical drinking day (i.e., count of drinks), and instances of past 30-day binge drinking (i.e., count of binges), defined as ≥5 drinks on a single occasion regardless of sex. From those variables, we derived a binary indicator of heavy drinking (any vs. none), defined as ≥3 drinks/day for males or ≥2 drinks per day for females following the recommended drinking limits at the time of the study [42], and a binary indicator of binge drinking (any vs. none). We created the heavy drinking and binge drinking indicators only for respondents who reported any current drinking as non-drinkers would have zero probability of these behaviors.

Four multiple-item scales assessing psycho-social experiences during the pandemic were drawn from the same collaborative survey noted above [41]. They served as our focal predictors and included emotional and physical reactions to the pandemic (e.g., “Since the breakout of the COVID-19 pandemic, I feel nervous, anxious, or on edge;” 12 items, α = 0.91); disruption of daily activities and social interactions due to the pandemic (e.g., “Since the breakout of the COVID-19 pandemic, I have had difficulty taking care of my children’s needs or balancing their needs with other responsibilities;” six items, α = 0.84); financial hardship (e.g., “Since the breakout of the COVID-19 pandemic, I have experienced financial difficulties;” four items, α = 0.84); and receiving or giving social support (e.g., “Since the breakout of the COVID-19 pandemic, I have received tangible support from family or friends when needed;” four items, α = 0.66). Respondents rated each item on a five-point Likert-type scale (strongly disagree to strongly agree). For analysis, we created a count variable of item endorsements (agree or strongly agree) within the scale. Higher counts indicated greater exposure to that pandemic-related experience (see Supplementary Materials for further details).

Finally, we included 11 demographic and health status variables to describe the sample and serve as potential control variables, including age, gender identity, race/ethnicity, sexual orientation, relationship status, educational attainment, employment status, rural residence, the presence of minor children in the household, self-rated health, and comorbid conditions. The variables were selected based on empirical evidence (i.e., factors known to be associated with alcohol use) and within the constraints of a secondary analysis (i.e., variables available in the parent study).

Missing data was minimal (range 0–4.5%) for nearly all variables and appeared random; therefore, we used complete case analysis. However, five indicators of pandemic-related experiences had higher missingness (range 6.1–9.6%). As the multiple-item measures of pandemic-related experiences showed strong internal consistency reliability, we chose not to impute any missing indicators when calculating summary scores. The analysis began by calculating unweighted summaries of demographic and health status variables to describe the sample. We then estimated the prevalence of alcohol use outcomes, accounting for the complex sampling design with survey weights. Next, we followed the model-building procedures described by Hosmer, Lemeshow, and Sturdivant [43]. Briefly, this was a multiple-stage, purposive strategy that relied on goodness-of-fit assessments (e.g., the likelihood ratio test) to determine whether the inclusion of a predictor improved the fit of a logistic regression model. Although the process began with bivariate tests, it was more sophisticated than single tests of association and served our goal to arrive at the best-fitting, most parsimonious model to explain each alcohol use outcome. We used the four pandemic-related experiences as focal predictor variables and the set of 11 demographic and health status variables as candidate control variables. Variables were not included in the final model if they failed to improve fit at any point in the model-building process. Again, we used survey weights to account for the sampling strategy and set the critical alpha for significance in final multiple variable models at 0.05. All analyses were performed in SAS (v9.4). The study was not pre-registered, and the data are not publicly available due to the risk of deductive identification of respondents (please see the Data Availability Statement below).

## 3. Results

Survey respondents’ demographic characteristics are summarized in Table 1. Majorities of the sample were White (96.0%), female (55.2%), married or co-habitating (71.6%), heterosexual (97.8%), and employed (52.4%). More than half the sample was age 60 years or older (55.4%) and had some college or a college degree (71.7%). A sizable minority lived in rural counties (38.6%).

**Figure 1:**
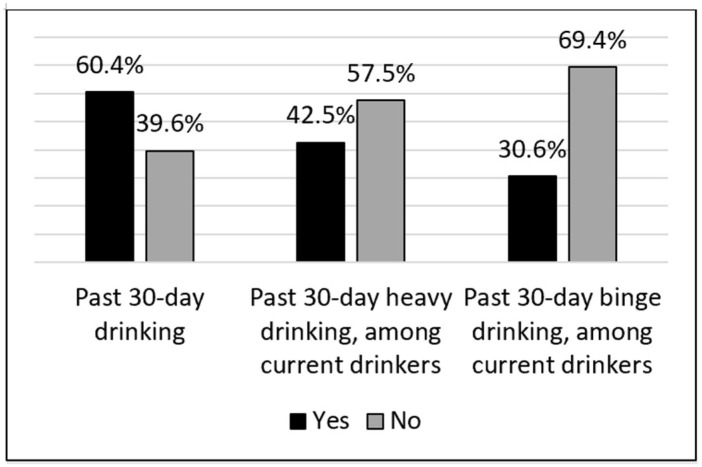
Self-reported past 30-day drinking in the first year of the COVID-19 pandemic. Notes: Percentages are weighted. Heavy drinking is defined as ≥3 drinks/day for men or ≥2 drinks per day for women. Binge drinking is defined as ≥5 drinks for both men and women.

More than one-third (39.6%) of respondents indicated no alcohol consumption in the past 30 days (Figure 1). Among the majority of respondents who were current drinkers, 42.5% disclosed heavy drinking in the past 30 days and 30.6% reported binge drinking in the past 30 days.

**Figure 2:**
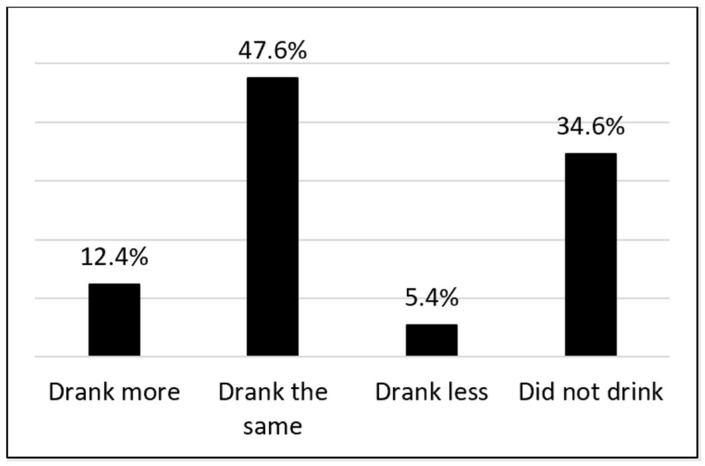
Self-reported changes in drinking since the onset of the COVID-19 pandemic. Note: Percentages are weighted.

Nearly half (47.6%) of the sample reported no change in drinking compared to before the pandemic (Figure 2). However, a minority of respondents (12.4%) reported increasing their drinking compared to before the pandemic, and a smaller minority (5.4%) reported decreasing their drinking compared to before the pandemic.

Table 2 shows the final regression models of each alcohol use outcome. Variables are absent from each column if they were not retained during the model-building process. Examining our primary outcome, two types of COVID-19 pandemic experiences and four demographic characteristics were associated with increased drinking during the first year of the pandemic. Emotional and physical reactions to the pandemic and disruptions of daily activities and social interactions due to the pandemic were associated with approximately 13% and 22% higher odds of increased drinking (adjusted odds ratio [aOR] 1.126 and aOR 1.223, respectively). In addition, educational attainment had an association with increased drinking, but only at the highest levels of education. Respondents with some college, vocational or technical school had 89% higher odds of increased drinking (aOR 1.886), while those with a college degree had more than twice the odds of increased drinking (aOR 2.334), compared to respondents whose education was less than a high school diploma. Age showed a mixed effect, with respondents in the 30–39 years category having 83% higher odds of increased drinking (aOR 1.832), but the oldest age category (70 years and older) had less than half the odds of increased drinking (aOR 0.449), compared to respondents in the 18–29 years category. Employment status had a limited protective association, such that being out of the labor force (i.e., homemaker, student, retired, or unable to work) was associated with 44% lower odds of increasing drinking (aOR 0.565) compared to respondents who were employed. Finally, rural residents showed an inverse association, with those respondents having 41% lower odds of increased drinking (aOR 0.594) compared to respondents who were not rural residents. Neither self-rated health nor comorbidities had any association with increased drinking.

We then examined two types of at-risk drinking, heavy drinking and binge drinking, among current drinkers. No pandemic experience was associated with heavy drinking, but three demographic characteristics showed associations. Age had a limited effect, with only the oldest stratum associated with 67% lower odds of heavy drinking (aOR 0.334) compared to respondents in the 18–29 years category. In contrast, gender identity and relationship status had positive associations. Female respondents had nearly twice the odds of heavy drinking than male peers (aOR 1.913), and formerly married and never married respondents had 69% and 71% higher odds of heavy drinking than married respondents (aOR 1.687 and aOR 1.706, respectively).

In terms of binge drinking, each one-unit increase in pandemic-related social support score was associated with 13% lower odds (aOR 0.867). Among demographic correlates, age had a limited effect, with only the two oldest strata associated with significantly lower odds of binge drinking (60–69 years, aOR 0.533; and 70+ years, aOR 0.256, respectively) compared to respondents in the 18–29 years category. Gender identity was associated with binge drinking, with female respondents having more than twice the odds of binge drinking compared to their male counterparts (aOR 2.122). In addition, relationship status had a significant association, such that formerly married and never married respondents had 70% and 63% higher odds of binge drinking compared to married peers (aOR 1.695 and aOR 1.625, respectively). Among health status variables, comorbidities had a limited effect. Compared to having no comorbidities, having one comorbid condition was associated with 41% higher odds of binge drinking (aOR 1.406).

## 4. Discussion

We conducted these analyses to extend COVID-19 pandemic knowledge by not only documenting changes in alcohol use in Iowa but also by assessing the associations of four specific experiences—three types of pandemic-related stressors and salient social support—with alcohol use. There have been many reports of increased drinking in response to the COVID-19 pandemic. To our knowledge, however, there have been only two previous research reports of changes in alcohol consumption in midwestern states during the COVID-19 pandemic, both from studies conducted in Wisconsin, a neighboring state [18,44]. In general, the results supported our hypotheses that there would be positive associations between pandemic-related stressors and alcohol use and a negative association between social support and alcohol use; however, the associations were not consistent across all alcohol use outcomes.

Although the proportion of survey respondents who reported increasing their alcohol use was modest (12.4%), it is consistent with US and international literature [13,16,17,25,38]. Pandemic-related experiences may have prompted the change in our sample. Drinking is a widely acknowledged coping response to stressors, and we identified two correlates of increased drinking—emotional and physical reactions to the pandemic, and disruption of daily activities and social interactions due to the pandemic. This suggests that some people may not have been well equipped to manage those stressors and could benefit from interventions to support healthy coping strategies. Building resilience before a catastrophic event (e.g., natural disaster, armed conflict, or disease outbreak) may have considerable long-term benefits. For example, disaster preparation campaigns could include education about healthy coping strategies, or emergency responses during a crisis could include prompts to utilize social networks to provide or seek support from others.

However, many respondents’ drinking did not change compared to before the pandemic, and a minority of our sample (5.3%) reduced their drinking. Similar mixed changes in alcohol use, including reductions in drinking, have been reported in the literature [15,18,19,45]. Understanding the rationale to reduce drinking may support novel health promotion interventions during similar large-scale crises. Unfortunately, the survey did not assess respondents’ drinking motivations or personality traits that might be associated with unhealthy coping (e.g., sensation seeking). Thus, we are limited in how much we can explain the changes in alcohol use in our sample.

Two other results are worth noting. First, rural residence was associated with substantially lower odds of increased drinking. This is in contrast to Holland and colleagues [19], who found greater odds of increased drinking among rural residents, and Czeisler and colleagues [46], who found no difference by rurality. We see this heterogeneity as a challenge to the conventional deficit framing of rural populations. There may be protective factors in rural contexts that could be leveraged for health promotion. These could include a strong shared identity, high social cohesion (i.e., norms of reciprocity, trust in others), and the availability of alternative civic organizations (e.g., grange halls, faith communities). Unfortunately, the survey did not collect further contextual information. Thus, we look forward to future research that adopts a comprehensive approach, investigating rural strengths and assets for health as well as needs. Second, higher education levels were associated with greater odds of increased drinking. As education typically functions as a protective factor against injurious health behaviors, we posit that our finding may be due to unanticipated secondary effects of education. Higher income could allow regular purchase of alcohol, or people with higher education levels may be employed in jobs that permit working from home, which could facilitate more frequent drinking. This is consistent with findings from US general population surveys that have found higher levels of regular drinking, and lower proportions of alcohol abstainers, among respondents with college degrees compared to peers without [47,48]. As the preponderance of research has focused on low educational attainment as a risk factor for hazardous drinking and alcohol-related problems, the mechanisms responsible for the associations detected here warrant further exploration. A better understanding of the ways that education facilitates alcohol use could inform future risk-reduction interventions.

We also investigated two secondary outcomes, heavy drinking and binge drinking, among current drinkers, finding largely null results. Against expectations, no pandemic-related stressor was associated with either outcome. This is in contrast to Rodriguez and colleagues [25], who found perceived COVID-19 threat was associated with heavy drinking. However, our findings are congruent with Yue and colleagues [45], who found that general stress—but not COVID-19-related stress—was associated with both increased drinking and binge drinking. However, social support appeared to function as hypothesized, albeit in a limited way. Social support was associated with lower odds of binge drinking but had no association with heavy drinking. Thus, the two types of high-risk alcohol use are likely driven by other factors than proximal stressors or social support.

Other findings may help clarify alcohol risk profiles. Notably, women had higher odds than men of both secondary drinking outcomes. This may reflect the national US trend in which the historical gender gap in alcohol use is closing, with women’s drinking more closely resembling that of men [49,50,51]. We believe this underscores the need for targeted alcohol risk-reduction interventions for women. In addition, two protective factors emerged. Age had an inverse association with both heavy and binge drinking (although only in the oldest strata), and marriage appeared to have a widely beneficial effect. People who were not married or co-habitating had consistently higher odds of both heavy and binge drinking. These findings may guide targeted alcohol risk-reduction efforts (i.e., for young and early middle-aged adults or those who are not in close relationships). Finally, one health variable had a curious association. People with one comorbid condition had higher odds of binge drinking than peers with no comorbidity. Due to the cross-sectional data, we are unable to discern the ordering. It is possible that binge drinking could lead to a co-occurring health problem, or conversely that a health problem could prompt binge drinking as a maladaptive coping response. Additional studies are needed to confirm and extend this finding.

This study benefited from some notable strengths, such as a large probability sample that included oversampling of rural residents and timely data collection in the first year of the pandemic. It also took place in a heavy-drinking midwestern US state, underscoring the importance of the context of this study. Nevertheless, our study should be considered in light of potential limitations. First, the cross-sectional design precludes causal inferences. However, our hypotheses were intended only to identify associations of pandemic experiences with alcohol outcomes, not to establish causality. In addition, although directionality cannot be assured, the presumed ordering should hold—the outcomes (e.g., increased alcohol use; heavy and binge drinking) are unlikely to be antecedents of the predictors (e.g., pandemic-related stressors; demographic characteristics). Second, respondent characteristics may have affected the results. For example, the sample was predominantly White, and all surveys were completed in English. Although it reflected the demographic profile of Iowa, the sample’s limited diversity likely reduced our ability to detect differences for other races and ethnicities. Additionally, the sample included a large proportion of older adults. It is possible that they responded differently to pandemic-related stressors than younger adults. We acknowledge that any limitations related to sample characteristics were likely driven by the use of voter registration lists as the sampling frame and the predominantly mail survey mode, both of which favored White race and older age. Furthermore, the use of post hoc survey weights minimized any potential distortion of results due to over-sampling of rural residents; however, it did not ensure that sample results were representative of the state population. Third, the data may be subject to response biases due to self-reporting. In particular, respondents’ incorrect recall of drinking before the pandemic may have led to misreporting of changes in alcohol use during the pandemic. Fourth, binge drinking was assessed using the male threshold of five or more drinks on an occasion for all respondents. As the recommended practice is to use sex-specific definitions, it is likely that we underestimated the prevalence of binge drinking among women, which is defined as four or more drinks on an occasion.

## 5. Conclusions

Some pandemic-related stressors were associated with increased drinking, but none were associated with heavy or binge drinking. Findings from our study can inform future responses to large-scale stressors, as a better understanding of both risk and resiliency factors can inform effective prevention interventions, policy decisions, and preparations for future catastrophic events. In addition, this study extends knowledge about alcohol consumption in Iowa, and findings may prompt further efforts to reduce excessive drinking as a population health priority outside of responses to a large-scale crisis.

## Figures and Tables

**Table 1:** Survey respondents’ demographic characteristics.

Characteristic	n	%
Age		
18–29 years	227	(5.7)
30–39 years	395	(9.9)
40–49 years	454	(11.4)
50–59 years	703	(17.6)
60–69 years	1007	(25.3)
70+ years	1199	(30.1)
Gender identity		
Female	2198	(55.2)
Male	1773	(44.6)
Transgender or other gender	8	(0.2)
Race/ethnicity		
White	3788	(96.0)
Black or African American	32	(0.8)
Asian or Asian American	37	(0.9)
American Indian or Alaska Native	33	(0.8)
Hispanic or Latino	57	(1.4)
Sexual orientation		
Heterosexual or straight	3735	(97.8)
Gay, lesbian or bisexual	83	(2.2)
Relationship status		
Married or co-habitating	2852	(71.6)
Widowed, divorced, or separated	751	(18.8)
Never married	383	(9.6)
Educational attainment		
Less than high school diploma	112	(2.8)
High school diploma or GED	1015	(25.5)
Some college, vocational or technical school	1218	(30.6)
College degree	1638	(41.1)
Employment status		
Employed	2081	(52.4)
Unemployed	89	(2.2)
Out of labor force ^a^	1802	(45.4)
Residence		
Rural	2487	(61.5)
Not rural	1560	(38.6)
Minor child in household	859	(21.9)
Self-rated health		
Excellent or very good	2038	(50.9)
Good, fair, or poor	1966	(49.1)
Comorbid health conditions		
None	1718	(42.5)
One	1251	(31.0)
Two	622	(15.4)
Three or more	451	(11.2)

^a^ Out of the labor force = homemaker, student, retired, or unable to work. Notes: Frequencies and proportions are unweighted. Due to item-missing data not all counts sum to the total sample size (n = 4047).

**Table 2:** Final multiple variable models of alcohol use outcomes.

	Increased Drinking (Full Sample: n = 4047)			Heavy Drinking (Current Drinkers: n = 2267)			Binge Drinking (Current Drinkers: n = 2267)		
	aOR	(95% CI)		aOR	(95% CI)		aOR	(95% CI)	
COVID-19 pandemic experiences									
Emotional reactions to pandemic	1.126	(1.076, 1.180)							
Disruptions due to pandemic	1.223	(1.110, 1.347)					0.931	(0.859, 1.008)	
Financial hardships due to pandemic	1.100	(0.957, 1.265)		1.025	(0.912, 1.151)		1.074	(0.946, 1.218)	
Social support	0.922	(0.822, 1.033)					0.867	(0.790, 0.953)	
Demographic characteristics									
Age									
18–29 years	ref.								
30–39 years	1.832	(1.013, 3.312)		0.916	(0.550, 1.523)		0.886	(0.522, 1.503)	
40–49 years	1.612	(0.882, 2.946)		0.934	(0.557, 1.565)		0.862	(0.505, 1.471)	
50–59 years	1.266	(0.694, 2.309)		0.735	(0.447, 1.207)		0.620	(0.368, 1.043)	
60–69 years	0.980	(0.523, 1.837)		0.657	(0.397, 1.087)		0.533	(0.314, 0.906)	
70+ years	0.449	(0.205, 0.985)		0.334	(0.188, 0.595)		0.256	(0.139, 0.472)	
Gender identity									
Male				ref.			ref.		
Female				1.913	(1.549, 2.362)		2.122	(1.700, 2.650)	
Transgender or other gender				1.288	(0.238, 6.979)		2.770	(0.338, 22.704)	
Sexual orientation									
Heterosexual or straight	ref.						ref.		
Gay, lesbian or bisexual	1.201	(0.632, 2.282)					0.687	(0.373, 1.267)	
Educational attainment									
Less than high school diploma	0.958	(0.124, 7.387)							
High school diploma or GED	ref.								
Some college, vocational or technical school	1.886	(1.138, 3.125)							
College degree	2.334	(1.441, 3.781)							
Relationship status									
Married or co-habitating	ref.			ref.			ref.		
Widowed, divorced, or separated	1.082	(0.735, 1.592)		1.687	(1.246, 2.284)		1.695	(1.252, 2.296)	
Never married	0.949	(0.600, 1.501)		1.706	(1.138, 2.558)		1.625	(1.066, 2.477)	
Employment status									
Employed	ref.			ref.			ref.		
Unemployed	1.054	(0.536, 2.072)		0.890	(0.426, 1.862)		0.989	(0.473, 2.067)	
Out of labor force ^a^	0.565	(0.379, 0.844)		0.926	(0.685, 1.251)		0.918	(0.676, 1.247)	
Rurality									
No	ref.								
Yes	0.594	(0.443, 0.796)							
Health status									
Self-rated health									
Excellent or very good	ref.								
Good, fair, or poor	0.784	(0.584, 1.053)							
Comorbidities									
None	ref.						ref.		
One condition	0.973	(0.715, 1.323)					1.406	(1.093, 1.807)	
Two conditions	0.679	(0.410, 1.123)					1.190	(0.822, 1.722)	
Three or more conditions	0.557	(0.274, 1.133)					1.253	(0.773, 2.030)	

aOR = adjusted odds ratio (i.e., accounting for sampling strategy); 95% CI = 95% confidence interval; Ref. = referent category for comparison to other levels of a categorical variable; ^a^ Out of the labor force = homemaker, student, retired, or unable to work.

## Data Availability

The dataset presented in this article is not readily available because of the risk of deductive identification of respondents. Requests to access the datasets should be directed to mary-charlton@uiowa.edu.

## Supplementary Materials

The following supporting information can be downloaded at: https://www.mdpi.com/article/10.3390/ijerph22081230/s1, File S1: Measures of Pandemic-Related Experiences.
