# Supplementary material for: COVID-19 Pandemic Experiences and Hazardous Alcohol Use: Findings of Higher and Lower Risk in a Heavy-Drinking Midwestern State
Source: Int J Environ Res Public Health. 2025 Aug 7;22(8):1230. doi: 10.3390/ijerph22081230 (PMC12386519; doi:10.3390/ijerph22081230)
Supplement: Supplementary file 1 [file ijerph-22-01230-s001.zip › ijerph-3741579-supplementary.pdf]

Supplementary. Measures of Pandemic-Related Experiences

Emotional and Physical Reactions

Since the breakout of the COVID-19 pandemic…

1. I feel nervous, anxious, or on edge.
2. I feel anxious about getting COVID-19.
3. I worry about possibly infecting others.
4. I am concerned about a family member or close friend getting or dying from COVID-19.
5. I worry about the possibility of dying from COVID-19.
6. I feel I have no control over how COVID-19 will impact my life.
7. I have experienced feelings of sadness or depression.
8. I feel negative about the future.
9. I have experienced changes in my sleep.
10. I have experienced changes in my eating.
11. I have experienced difficulty concentrating.
12. I have experienced feelings of social isolation or loneliness.

Disruption to Daily Activities and Social Interactions

Since the breakout of the COVID-19 pandemic…

1. I have experienced disruptions in day-to-day activities with family and/or friends.
2. I have had trouble adequately taking care of family members or friends I provide for.
3. I have been unable to follow my typical daily routines (e.g., work, exercise, leisure activities).
4. I have experienced conflict with household members (e.g., spouse/partner, children, parents, others).
5. I have had difficulty or been unable to do my work as usual.
6. I have had difficulty taking care of my children's needs (e.g. providing care, supervising schoolwork), and/or balancing their needs with other responsibilities.

Financial Hardship

Since the breakout of the COVID-19 pandemic…

1. I have experienced financial difficulties.
2. I have had difficulty purchasing or obtaining basic necessities (e.g., food, personal care products).
3. I feel anxious about losing or having lost my job, or my primary source of income.
4. I have been unable to adequately provide for people I financially support.

Social Support

Since the breakout of the COVID-19 pandemic…

1. I have received emotional support from family or friends when needed.
2. I have received tangible support (e.g., financial, practical) from family or friends when needed.
3. I am or have been there to listen to others' problems when needed.
4. I have helped others with financial or practical support.

Response options = strongly disagree; disagree; neutral; agree; strongly agree; not applicable
